# Post Mortem Examinations, with Special Reference to Medico-Legal Practice

**Published:** 1880-05

**Authors:** 


POST MORTEM EXAMINATIONS, WITH SPECIAL REFERENCE TO MEDICO-LEGAL PRACTICE. By Prof. Rudolph Virchow, of the Berlin Charite Hospital. Translated from the second German edition by Dr. T. P. Smith. Philadelphia: PRESLEY BLAKISTON, 1012 Walnut st. 1880.

This is a neat little volume of 145 pages, and will doubtless be found interesting and beneficial.

